# Identification of *Aeromonas hydrophila* Genes Preferentially Expressed after Phagocytosis by *Tetrahymena* and Involvement of Methionine Sulfoxide Reductases

**DOI:** 10.3389/fcimb.2016.00199

**Published:** 2016-12-26

**Authors:** Maoda Pang, Xiaoqin Lin, Jin Liu, Changming Guo, Shanshan Gao, Hechao Du, Chengping Lu, Yongjie Liu

**Affiliations:** ^1^Department of Preventive Veterinary, College of Veterinary Medicine, Nanjing Agricultural UniversityNanjing, China; ^2^Key Lab of Food Quality and Safety of Jiangsu Province-State Key Laboratory Breeding Base, Institute of Food Safety, Jiangsu Academy of Agricultural SciencesNanjing, China

**Keywords:** *Aeromonas hydrophila*, *Tetrahymena*, phagocytosis, SCOTS, *msr* genes

## Abstract

Free-living protozoa affect the survival and virulence evolution of pathogens in the environment. In this study, we explored the fate of *Aeromonas hydrophila* when co-cultured with the bacteriovorous ciliate *Tetrahymena thermophila* and investigated bacterial gene expression associated with the co-culture. Virulent *A. hydrophila* strains were found to have ability to evade digestion in the vacuoles of this protozoan. In *A. hydrophila*, a total of 116 genes were identified as up-regulated following co-culture with *T. thermophila* by selective capture of transcribed sequences (SCOTS) and comparative dot-blot analysis. A large proportion of these genes (42/116) play a role in metabolism, and some of the genes have previously been characterized as required for bacterial survival and replication within macrophages. Then, we inactivated the genes encoding methionine sulfoxide reductases, *msrA*, and *msrB*, in *A. hydrophila*. Compared to the wild-type, the mutants Δ*msrA* and Δ*msrAB* displayed significantly reduced resistance to predation by *T. thermophila*, and 50% lethal dose (LD_50_) determinations in zebrafish demonstrated that both mutants were highly attenuated. This study forms a solid foundation for the study of mechanisms and implications of bacterial defenses.

## Introduction

*Aeromonas hydrophila*, a Gram-negative ubiquitous bacterium with diverse host specificity, is distributed widely in aquatic environments (Daskalov, 2006; Janda and Abbott, 2010). *Aeromonas* infection has been linked to major die-offs and fish kills and has thus resulted in significant economic losses around the world for decades (Pang et al., 2015). In addition, this bacterium has been proposed to cause a variety of serious illnesses in other cold-blooded species and humans (Janda and Abbott, 2010). The pathogenesis of *A. hydrophila* is multifactorial and is likely mediated by virulence factors such as adhesins, exotoxins, extracellular enzymes, secretion systems, iron acquisition systems, and quorum-sensing systems (Tomas, 2012). Notably, environmental factors, such as predation by heterotrophic protists, have a dramatic effect on the virulence evolution of pathogens (Erken et al., 2013). However, the mechanism underlying this has not been investigated in *A. hydrophila*.

*A. hydrophila* can be isolated from numerous aquatic environments, such as drinking water, groundwater, wastewater, rivers, lakes, ponds, and sewage in various stages of treatment (Janda and Abbott, 2010). The free-living ciliate *Tetrahymena* is commonly found in the same aquatic environments (Valster et al., 2009). Evidence increasingly supports interactions between *Tetrahymena* and microbial pathogens. King et al. (1988) reported that many bacterial pathogens can resist the grazing protozoan *Tetrahymena pyriformis*. After predation by *Tetrahymena* species, *Legionella pneumophila* (Berk et al., 2008; Hojo et al., 2012) and *Salmonella enterica* (Brandl et al., 2005) are released in a viable form in vesicles or pellets from the protozoa. Due to the presence of a membrane around the vesicle, the bacterial cells within the vesicles are more resistant to disinfectants than those remaining free in suspension (Brandl et al., 2005). Ciliates thus may act as a reservoir for potentially pathogenic bacteria (Brandl et al., 2005). Grazing by phagotrophic protists is an important course of microbial mortality in aquatic environments (Pernthaler, 2005). To resist this predation, virulence factors in many bacterial species may have evolved for anti-predator defense (Ahmed et al., 2010; Erken et al., 2013).

Rahman et al. (2008) indicated that amoebae present in aquatic environments play an important role as reservoirs for *Aeromonas* species. We have previously demonstrated that the hypervirulence phenotype of *A. hydrophila* can survive efficiently within *T. thermophila* (Li et al., 2011; Pang et al., 2012). All of this evidence indicates an important link between *Aeromonas* and grazing protozoa. The question then arose as to which bacterial genes were involved in the anti-predator defense. In this study, we investigated the fate of *A. hydrophila* strains after co-culture with *T. thermophila* and used selective capture of transcribed sequences (SCOTS) to identify the genes that were preferentially expressed by *A. hydrophila* upon interaction with this protozoan. Additionally, we evaluated the role of the *msr* genes of *A. hydrophila*, which encode methionine sulfoxide reductases, in the response to predation by *T. thermophila*.

## Materials and methods

### Strains and culture conditions

Seven virulent *A. hydrophila* strains (NJ-35, XY-16, NJ-34, CS-43, NJ-1, XX-14, and NJ-37), and five avirulent *A. hydrophila* strains (NJ-28, JH-19, NJ-3, CS-34, and JH-17; Pang et al., 2012), were used in this study (Table 1). The nucleotide sequence of the complete genome of NJ-35 has been deposited in GenBank (accession number CP006870). The bacterial strains were routinely cultured in Luria broth (LB) containing 1% NaCl, 1% peptone, and 0.5% yeast extract at 28°C. *T. thermophila* SB210 (Eisen et al., 2006) was obtained from Dr. Miao Wei, Institute of Hydrobiology, China Academy of Sciences. The genome sequence of *T. thermophila* SB210 has been deposited in GenBank under accession number GCA_000261185.1. *T. thermophila* SB210 was grown axenically in SPP medium (2% protease peptone, 0.1% yeast extract, 0.2% glucose, 0.003% EDTA-Fe) at 28°C and maintained in 5 mL of ultrapure water containing soybean. *A. hydrophila* and *T. thermophila* were co-cultured in TBSS (2 mM KCl, 1 mM CaCl_2_, 0.5 mM MgCl_2_,and 1 mM Tris [pH 6.8–7.2]). All reagents used in this study were supplied by Sigma (St. Louis, MO, USA) unless otherwise indicated.

**Table 1:** **Strains and plasmids used in this study**.

**Strain, plasmid, or primer**	**Characteristic and/or sequence (5′–3′)**	**Source/references**
**STRAIN**		
NJ-35	Virulent wild-type *A. hydrophila* strain, Amp^r^	Pang et al., 2012
XY-16	Virulent wild-type *A. hydrophila* strain, Amp^r^	Pang et al., 2012
NJ-34	Virulent wild-type *A. hydrophila* strain, Amp^r^	Pang et al., 2012
CS-43	Virulent wild-type *A. hydrophila* strain, Amp^r^	Pang et al., 2012
NJ-1	Virulent wild-type *A. hydrophila* strain, Amp^r^	Pang et al., 2012
XX-14	Virulent wild-type *A. hydrophila* strain, Amp^r^	Pang et al., 2012
NJ-37	Virulent wild-type *A. hydrophila* strain, Amp^r^	Pang et al., 2012
NJ-28	Avirulent wild-type *A. hydrophila* strain, Amp^r^	Pang et al., 2012
JH-19	Avirulent wild-type *A. hydrophila* strain, Amp^r^	Pang et al., 2012
NJ-3	Avirulent wild-type *A. hydrophila* strain, Amp^r^	Pang et al., 2012
CS-34	Avirulent wild-type *A. hydrophila* strain, Amp^r^	Pang et al., 2012
JH-17	Avirulent wild-type *A. hydrophila* strain, Amp^r^	Pang et al., 2012
SM10	*E. coli* strain, λpir^+^, Kan^r^	Park et al., 2004
Δ*msrA*	*msrA* gene deletion mutant of NJ-35, Amp^r^	This study
Δ*msrB*	*msrB* gene deletion mutant of NJ-35, Amp^r^	This study
Δ*msrAB*	*msrA* and *msrB* double genes deletion mutant of NJ-35, Amp^r^	This study
CΔ*msrA*	Δ*msrA* complemented with pMMB-*msrA*, Amp^r^,Cm^r^	This study
CΔ*msrB*	Δ*msrB* complemented with pMMB-*msrB*, Amp^r^,Cm^r^	This study
**PLASMID**		
pYAK1	R6K-*ori* suicide vector, SacB^+^, Cm^r^	Abolghait, 2013
pYAK-*msrA*	Plasmid pYAK1 carrying the flanking sequences of *msrA*	This study
pYAK-*msrB*	Plasmid pYAK1 carrying the flanking sequences of *msrB*	This study
pMMB207	Low-copy-number vector, Cm^r^	Morales et al., 1991
pMMB-*msrA*	Plasmid pMMB207 carrying the complete ORF of *msrA*	This study
pMMB-*msrB*	Plasmid pMMB207 carrying the complete ORF of *msrB*	This study
pWSK129-*gfp*	Green fluorescent protein (GFP) expression	Li et al., 2011
pMD18-T	Vector for cloning Taq polymerase-amplified PCR products	TaKaRa
pMD18-T16S	pMD18-T carrying the 16S rRNA sequence (1537bp) of strain NJ-35	This work
pMD18-T23S1	pMD18-T carrying the 5′ end 1440 bp fragment of 23S rRNA of strain NJ-35	This work
pMD18-T23S2	pMD18-T carrying the 3′ end 1377 bp fragment of 23S rRNA of strain NJ-35	This work

### Survival of *A. hydrophila* in *T. thermophila* vacuoles

To track the survival of *A. hydrophila* in *T. thermophila*, seven virulent strains and five avirulent strains were intrinsically labeled with green fluorescent protein (GFP) by electroporation of the plasmid pWSK129-*gfp* (Li et al., 2011). Then, 5000:1 co-cultures of *A. hydrophila* and *T. thermophila* were used to investigate their interaction (Pang et al., 2012). Before co-culture, *T. thermophila* SB210 with an initial inoculum of 10^3^ cells/mL was grown in 50 mL of SPP medium at 28°C for 36 h, when the cultures entered stationary phase. The cells were washed twice with TBSS, counted using a hemacytometer, and then diluted in TBSS to a concentration of 2 × 10^5^ cells/mL. *A. hydrophila* was incubated in 5 mL of LB medium at 28°C for 12 h until stationary-phase growth using an initial inoculum of 10^7^ cells/mL, washed twice with TBSS, and then adjusted to 1 × 10^9^ CFU/mL using TBSS. Five hundred microliters of *A. hydrophila* suspension was mixed with an equal volume of *T. thermophila* cells and incubated at 28°C for 12 h without shaking. The bacterial cells in *T. thermophila* were observed by laser scanning confocal microscopy (LSCM, Zeiss LSM710). In addition, co-cultures were prepared for transmission electron microscopy (TEM, Hitachi H-7650) by pelleting the cells and immediately fixing them with 2.5% glutaraldehyde (Solarbio, Beijing, China) for 2 h at 4°C. TEM observation was performed as described by Serratrice et al. (2014).

The LIVE/DEAD BacLight Bacterial Viability Kit (Invitrogen, New York, USA) was used to measure the proportion of viable bacterial cells contained in the vacuoles of *T. thermophila*. Propidium iodide (PI, Molecular Probes) was added at a final concentration of 7.5 μM to each of three replicate tubes containing the cultures, and the tubes were incubated for 15 min at 28°C in the dark. Then, the cultures were washed twice in TBSS and fixed in 2.5% glutaraldehyde. Ten microliters of each replicate suspension was placed on slides and observed by LSCM using the GFP and PI channels. For each sample, 100 vacuoles were examined, and the numbers of green (viable) and red (dead) fluorescent bacterial cells per vacuole were counted. Vacuoles were examined from three replicate tubes.

### Experimental infection, RNA extraction, cDNA synthesis, and amplification

For RNA extraction, 1 × 10^5^ *T. thermophila* cells were co-incubated with 5 × 10^8^ CFU of *A. hydrophila* NJ-35 without shaking for 12 h at 28°C. This time point was selected on the basis of our previous study, which demonstrated that virulent *A. hydrophila* strains survived better than avirulent *A. hydrophila* strains when co-cultured with *T. thermophila*, particularly after co-culture for 12 h (Pang et al., 2012). In considering the MOI, our preliminary dose-response studies showed that, the number of *A. hydrophila* which was taken up by an average protis nearly reached saturation at the MOI of 5000 (data not shown). After incubation, the co-cultures were centrifuged at 100 g for 1 min. Then, the obtained pellet was washed twice with TBSS by centrifugation at 200 g for 1 min and 400 g for 1 min, respectively. The final pellet was resuspended in 1 mL of TBSS and further treated with gentamycin (100 μg/mL) for 1 h to kill the remaining extracellular or adherent bacteria. Samples were then centrifuged at 400 *g* for 1 min and the harvested *Tetrahymena* cells were washed once in TBSS. The *Tetrahymena* pellet was then resuspended in 1 mL TBSS containing 1% Triton X-100 for 10 min at 37°C to release ingested bacteria. The suspension containing lysed *Tetrahymena* cells was centrifuged at 4000 g for 5 min at 4°C to provide the ingested bacteria. Control bacteria without *Tetrahymena* were incubated in TBSS for the same time, and then collected by centrifugation to provide protozoa-unexposed bacterial cells. Total RNA was extracted using TRIzol reagent (Invitrogen) from two samples containing equal numbers of *A. hydrophila* differing only in the presence (protozoa-exposed RNA) or absence (protozoa-unexposed RNA) of *T. thermophila*. The RNA was subsequently treated with DNase I (Fermentas) for 1 h at 37°C. The integrity, purity and concentration of the RNA were determined by agarose gel electrophoresis, PCR and A260/A280 spectrophotometer readings, respectively. The total RNA isolated from protozoa-exposed or protozoa-unexposed bacteria was converted to first-strand cDNA by random priming with Superscript II reverse transcriptase (Invitrogen) according to the manufacturer's specifications. The primers had a defined 5′ terminal sequence and a 3′ random hexamer, and different terminal sequences were used for protozoa-exposed (SCOTS-N6-01) and protozoa-unexposed RNA (SCOTS-N6-02; Froussard, 1992). The second strand of cDNA was synthesized using Klenow fragment (Fermentas). Then, the cDNA libraries were amplified by PCR with 25 cycles of amplification (95°C for 30 s, 66°C for 60 s, and 72°C for 60 s).

### Selective capture of transcribed sequences (SCOTS)

Bacterial transcripts were then separated from host cDNA by SCOTS as described previously (Guo et al., 2014). Briefly, denatured, biotinylated, and sonicated *A. hydrophila* genomic DNA (gDNA) fragments (0.6 μg) were mixed with 5 μg of sonicated ribosomal DNA (from plasmid pMD18-T16S, pMD18-T23S1, and pMD18-T23S2) to pre-block rRNA encoding regions on the gDNA. For each round of SCOTS, a sample of the mixture (8 μL) was denatured by incubation at 98°C for 3 min. The mixture was incubated at 64°C for 30 min, and 2 μL of 1 M NaCl was then added. At the same time, 2 μL of 1 M NaCl was added to the total amplified cDNA of bacteria exposed or unexposed to *T. thermophila* in 8 μL of 10 mM EPPS-1 mM EDTA. The denatured cDNA mixture was added to the biotinylated gDNA–rDNA pre-hybridized mixture, and hybridization was continued at 64°C for 24 h. Bacterial cDNA that was hybridized to biotinylated gDNA was then captured by binding hybrids to streptavidin-coated magnetic beads (Dynal M280). The captured cDNA was eluted, precipitated, and amplified by PCR using the defined primers SCOTS01 (protozoa-exposed) or SCOTS02 (protozoa-unexposed). For each condition, three rounds of capture were performed, and the normalized cDNA was obtained. The primers used in this study are showed in Supplementary Table 1.

### Competitive enrichment

To preferentially enrich for protozoa-exposed expressing transcripts, enrichment of cDNA was conducted to capture hybridizations. A total of 0.6 μg of *A. hydrophila* NJ-35 chromosome was pre-blocked with both 5 μg of rDNA and 5 μg of denatured triple-SCOTS normalized protozoa-unexposed cDNA. Then, 5 μg of triple-SCOTS normalized protozoa-exposed cDNA was denatured and re-annealed for 30 min at 64°C to remove abundant transcripts. The cDNA and blocked gDNA samples were combined and hybridized for 20 h at 64°C. Hybrids were collected using Dynal streptavidin-coated magnetic beads. The captured cDNA was eluted, precipitated, and amplified using the protozoa-exposed library-specific defined primer SCOTS01. After three rounds of this enrichment procedure, the cDNAs were ligated into the pMD18-T vector (TaKaRa, Dalian, China).

### Southern hybridization for primary verification and sequence analysis

To eliminate false-positive sequences that escaped the subtraction process, southern hybridization was used for primary verification. Cloned inserts obtained from protozoa-exposed-specific cDNA libraries were amplified by PCR with SCOTS01 primers. PCR amplicons of positive SCOTS clones were transferred to a positively charged membrane (Roche, Mannheim, Germany). Samples of gDNA and cDNA mixtures generated from protozoa-exposed strain NJ-35 and protozoa-unexposed strain NJ-35 were used as probes, followed by labeling with DIG-dUTP (Roche). Dot blot hybridization analysis using DIG Easy Hyb (Roche) was performed according to the manufacturer's instructions. The clones that hybridized positively with the protozoa-exposed probes but negatively with the protozoa-unexposed probes were termed SCOTS clones. Then, the inserts of positive cDNA clones were sequenced by GENEWIZ, Inc., and the nucleotide sequences were queried using BLASTn implemented in BLAST+ (version 2.2.29; ftp://ftp.ncbi.nlm.nih.gov/blast/executables/blast+/) against the genome of *A. hydrophila* NJ-35. To classify the functions of the preferentially expressed genes, BLASTp implemented in BLAST+ (version 2.2.29) was used to align the amino acid sequences against the COGs database (updated 2014), and some genes related to bacterial virulence were classified according to a previous study (Pang et al., 2015).

### Secondary verification using quantitative reverse transcription-PCR (qRT-PCR)

To further validate the SCOTS results, we randomly selected 14 genes to measure the level of expression by qRT-PCR. RNA extraction from protozoa-exposed strain NJ-35 and protozoa-unexposed strain NJ-35 was performed as described above. The altered expression levels of 14 genes in protozoa-exposed strain NJ-35 and protozoa-unexposed strain NJ-35 were examined individually. The cDNA was synthesized in triplicate using Superscript II with random hexamers (Invitrogen) according to the manufacturer's instructions. The QuantiTect SybrGreen PCR kit (Qiagen, Valencia, USA) was used for qRT-PCR in an ABI PRISM 7300 Fast Real-time PCR machine. For each sample, a no-reverse transcription reaction was performed as a no template control (NTC). The primers used are described in Supplementary Table 1. For each qRT-PCR run, the calculated cycle threshold (CT) was normalized to the CT of the internal control 16S rDNA amplified from the corresponding sample, and the fold-change was calculated using the 2^−ΔΔCT^ method as previously described (Livak and Schmittgen, 2001).

### Inactivation and complementation of *msrA* and *msrB* in *A. hydrophila*

The *msrA* mutant (Δ*msrA*) was constructed via homologous recombination using the suicide plasmid pYAK1. Briefly, the primers *msrA*-up-F/*msrA*-up-R and *msrA*-down-F/*msrA*-down-R were designed to amplify two flanking sequences of the *msrA* gene by PCR. Then, the two segments were ligated by fusion PCR and inserted into pYAK1 to construct the recombinant plasmid pYAK-*msrA* using *Escherichia coli* SM10 as the host strain. Subsequently, parental mating was used to transfer the recombinant plasmid pYAK-*msrA* into strain NJ-35 (Amp^r^). The transconjugants with the first allelic exchange were selected on LB agar plates with ampicillin and chloramphenicol. Positive clones were transferred to LB for growth for 12 h and then transferred to LB agar plates containing 10% sucrose. The suspected Δ*msrA* strain was verified by PCR. Using the same approach, the *msrB* deletion mutant and a double gene (*msrA, msrB*) deletion mutant were also constructed.

To complement the function of the deleted genes in the mutants, the complete ORFs of *msrA* and *msrB* were amplified from *A. hydrophila* genomic DNA to construct the pMMB-*msrA* and pMMB-*msrB* plasmids for genetic complementation. Then, the plasmids were introduced into Δ*msrA* and Δ*msrB* by conjugation using *E. coli* SM10 as the donor strain, and the complemented mutants CΔ*msrA* and CΔ*msrB* were selected on LB agar containing 100 μg/mL ampicillin and 34 μg/mL chloromycetin. The primers used for mutant construction are showed in Supplementary Table 1.

### Assessment of bacterial resistance to predation by *T. thermophila*

Bacterial resistance to predation was assessed by measuring the relative survival of bacteria after co-culture with *T. thermophila* (Pang et al., 2012). Briefly, *T. thermophila* SB210 was cultured at 28°C for 36 h in SPP medium until the stationary phase of growth using an initial inoculum of 10^3^ cells/mL. Cells were diluted in TBSS to a concentration of 2 × 10^5^ cells/mL. *A. hydrophila* was incubated in LB medium at 28°C for 12 h, washed twice with TBSS, and then adjusted to 1 × 10^9^ CFU/mL using TBSS. Five hundred microliters of *A. hydrophila* suspension was mixed with the same volume of *T. thermophila* cells, and 200 μl of these mixed cell suspensions was transferred into each well of a 96-well plate. *A. hydrophila* suspensions and *T. thermophila* suspensions mixed with an equal volume of TBSS separately served as controls. TBSS served as the blank control. Plates were incubated for 12 h at 28°C without shaking, and the bacterial population was detected by measuring the absorbance at 450 nm (OD450) every 2 h. The absorbance of *T. thermophila* cells was negligible (Pang et al., 2012). The relative survival of bacteria was expressed as the OD450 value of bacteria remaining in co-culture with *T. thermophila* divided by that of bacteria grown alone at 12 h. Three independent measurements were performed in quadruplicate.

### Determination of 50% lethal dose (LD_50_) in zebrafish

Zebrafish weighing ~3 g were supplied by Pearl River Fishery Research Institute, Chinese Academic of Fishery Science. The animal-challenge experiment with *A. hydrophila* was performed as described previously (Pang et al., 2012). For each *A. hydrophila* strain, eight groups of 15 zebrafish were intraperitoneally injected with 0.02 mL of 10-fold serially diluted suspensions of bacteria (10^1^–10^7^ CFU) in PBS. Another 15 zebrafish (the control group) were injected with 0.02 mL of sterile PBS. The survival rates of the zebrafish were recorded daily for a period of 7 days post infection, and the LD_50_ values were calculated. Animal experiments were conducted according to animal welfare standards and approved by the Ethical Committee for Animal Experiments of Nanjing Agricultural University, China.

### Statistical analysis

Data were collected and analyzed using MS Excel 2010 and SPSS Statics v20.0 software. Relative survival of bacteria was analyzed by analysis of variance (ANOVA) followed by Turkey's multiple comparison test; The gene expression levels in protozoa-exposed *A. hydrophila* and protozoa-unexposed *A. hydrophila* were analyzed using a Student's *t*-test; *P* < 0.05 was considered a significant difference, whereas *P* < 0.01 was considered highly significant.

## Results

### Survival of *A. hydrophila* in *T. thermophila*

**Figure 1:**
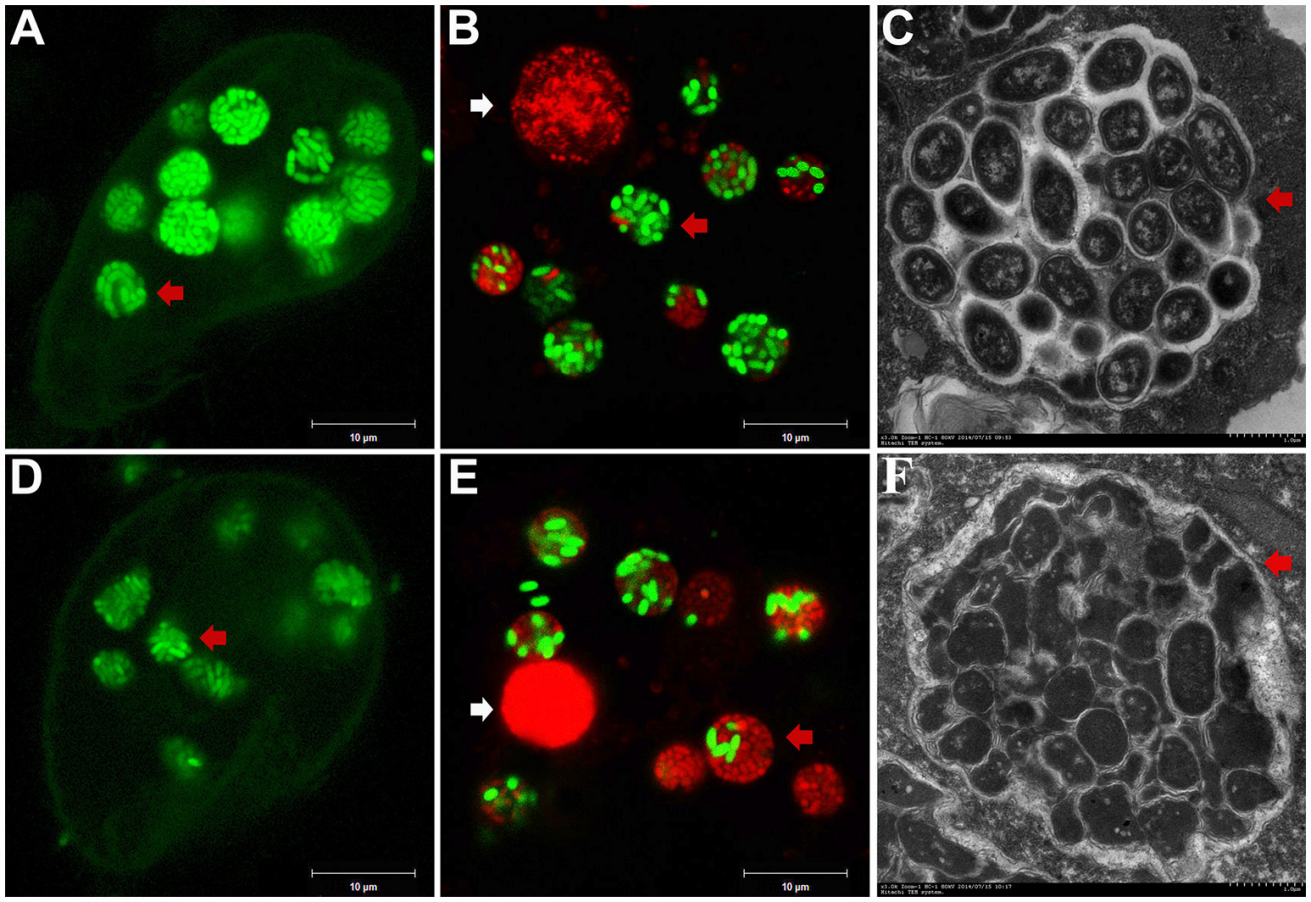
**Survival of *A. hydrophila* in *T. thermophila* vacuoles after co-culture for 12 h. (A–C)** show micrographs of virulent strain NJ-35 in *T. Thermophila*, and **(D–F)** show the micrographs of avirulent strain CS-34. **(A,D)** were acquired by LSCM (Zeiss LSM710) using a GFP channel and displayed by Gamma 0.45; **(B,E)** were acquired by LSCM (Zeiss LSM710) using GFP and PI channels; **(C,F)** were acquired by TEM (Hitachi H-7650). Viable and dead cells exhibit green and red fluorescence, respectively. The red arrow indicates bacterial cells in *T. thermophila* vacuoles. The white arrow indicates the nuclei of *T. thermophila* displaying red fluorescence when labeled by propidium iodide.

To investigate the fate of *A. hydrophila* in response to phagocytosis by *T. thermophila*, 12 *A. hydrophila* strains of different virulence were intrinsically labeled with GFP by transformation with the plasmid pWSK129-*gfp*. Then, LSCM was used to examine the predation of *A. hydrophila* by *T. thermophila* SB210. After the addition of bacteria to the *T. thermophila* suspensions, green food vacuoles could be observed in nearly all *T. thermophila* cells within 30 min, and *T. thermophila* fed readily on all *A. hydrophila* strains. Here, virulent strain NJ-35 and avirulent strain CS-34 were described as examples. As shown in Figure 1, after co-culture for 12 h, a high proportion of the cells of strain NJ-35 maintained their integrity and exhibited bright green fluorescent (Figure 1A), while strain CS-34 presented dispersed green fluorescent (Figure 1D).

To further analyze bacterial survival in vacuoles, GFP fluorescence in combination with PI viability staining was used in this study. GFP and PI exhibited good segregation of fluorescent labels in a mixed population of viable (green) and dead (red) cells. However, because the bacterial cells used in this study were labeled with GFP, some of the cells were yellow because of simultaneous red and green fluorescence, consistent with a previous study (Brandl et al., 2005). Such cells were relatively few (no more than 3%) and were not included in the counts. Compared to strain CS-34 (Figure 1E), more viable bacterial cells of strain NJ-35 (Figure 1B) were observed when co-cultured with *T. thermophila*. In addition, TEM observations also revealed that the intracellular NJ-35 remained morphologically intact (Figure 1C), whereas most of the intracellular CS-34 exhibited an irregular shape (Figure 1F).

**Figure 2:**
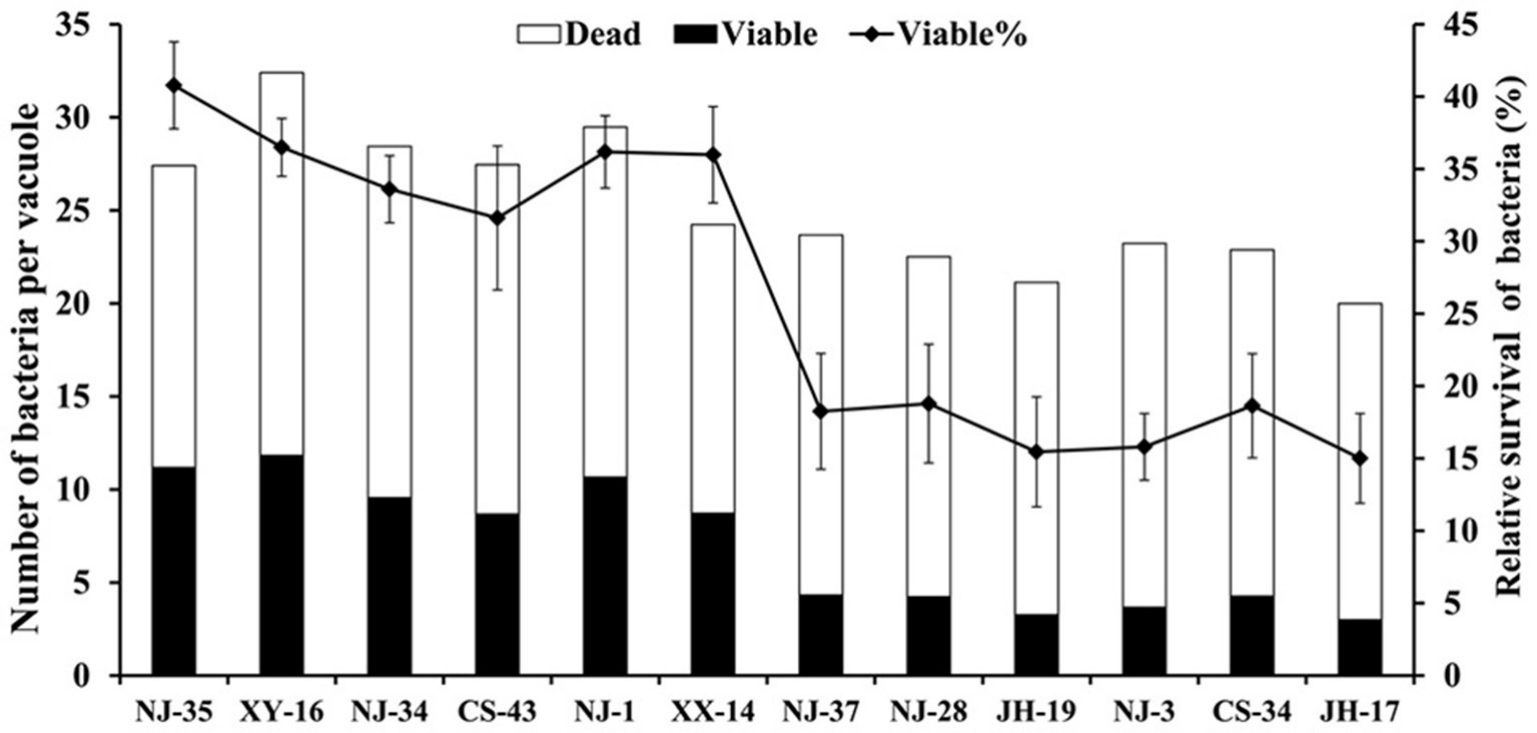
**Survival rate of *A. hydrophila* strains with different virulence in *T. thermophila* vacuoles**. Viable and dead bacterial cells in 100 *T. thermophila* vacuoles were counted using Zeiss LSM710, and the survival rate of the bacteria was expressed as the number of viable bacteria divided by the number of total bacteria per vacuole.

To support the speculation that virulent *A. hydrophila* strains may be able to evade digestion in the vacuoles of *T. thermophila*, the survival rates of seven virulent strains (NJ-35, XY-16, NJ-34, CS-43, NJ-1, XX-14, and NJ-37) and five avirulent strains (NJ-28, JH-19, NJ-3, CS-34, and JH-17) in vacuoles were calculated. As shown in Figure 2, after 12 h of co-culture, the survival rates of bacterial cells per vacuole in the virulent *A. hydrophila* groups, except strain NJ-37, were all higher than those of avirulent *A. hydrophila* groups. These findings indicated that virulent *A. hydrophila* strains may have a better ability to evade digestion in *T. thermophila* vacuoles.

### Selective capture of *A. hydrophila* transcripts

**Figure 3:**
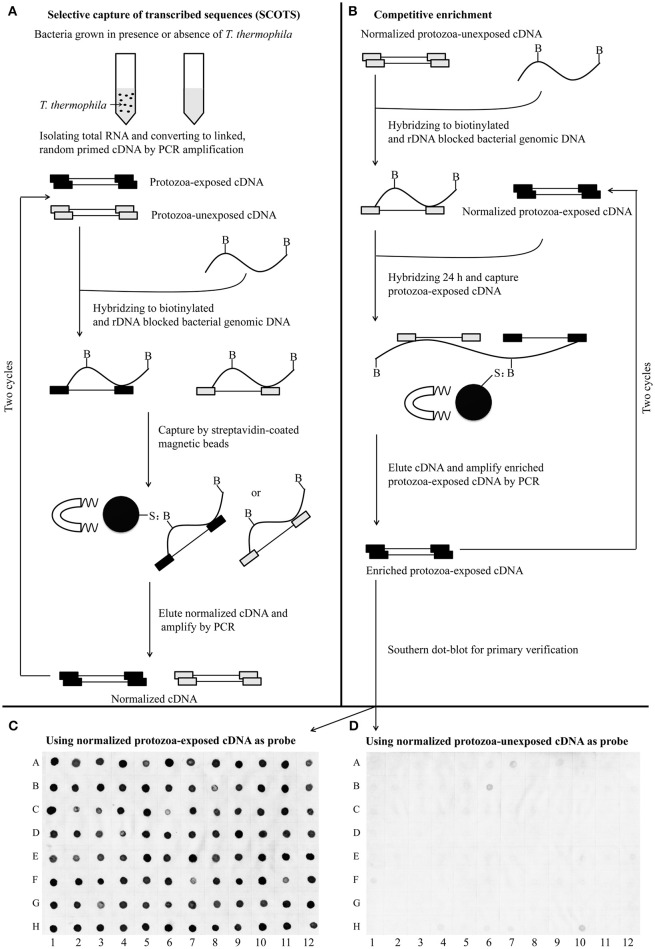
**Schematic presentation of the SCOTS approach followed by Southern dot-blot analysis. (A)** Normalization of protozoa-exposed cDNA and protozoa-unexposed cDNA; **(B)** competitive enrichment of protozoa-exposed expressing transcripts; **(C,D)** Southern dot-blot analysis of SCOTS clones using probes generated from normalized protozoa-exposed cDNA and protozoa-unexposed cDNA, respectively. The schematic presentations **(A,B)** were designed as described by An and Grewal (2012), with some modifications.

For identification of the genes that are differentially expressed by *A. hydrophila* NJ-35 when grown in protozoa-exposed and protozoa-unexposed environments, SCOTS (Figures 3A,B) was used in this study. After primary verification by southern hybridization (Figures 3C,D), a total of 288 positive SCOTS clones in the protozoa-exposed group were obtained and subjected to further sequence analysis. Subsequently, 256 available sequences were obtained. Among the 256 sequences, 26 sequences were unidentifiable “junk” DNA, and the remaining 230 sequences were identified as 116 genes since some of the sequences were the same. As shown in Table 2, these 116 genes were characterized into five functional categories: (1) Forty-two genes were involved in metabolism, such as amino acid transport, inorganic ion transport, energy production, carbohydrate transport, and metabolism. Genes such as *panF, gltD, oppA, purF, napA, thyA, mgtA, cysE*, and *norV* may endow the bacteria with ability to uptake multiple forms of nutrients or similar metabolites; (2) Twenty-two genes, including *rstB, msrA, msrB, clpP*, and *clpA*, encoded proteins responsible for cellular processes and signaling, including cell membrane biogenesis, post-translational modification, and signal transduction mechanisms; (3) Twenty genes, including *dnaA* and *rpoC*, were involved in information storage and processing, including transcription, replication, recombination, and repair; (4) Eighteen genes encoded proteins that can be characterized as virulence-associated factors, such as the type 6 secretion system (T6SS) effector proteins hemolysin co-regulated protein (Hcp) and valine glycine repeat G (VgrG), and proteins involved in motility and adhesion; (5) The remaining 14 genes were poorly characterized, and eight encoded hypothetical proteins.

**Table 2:** **Genes identified by SCOTS that were differentially expressed in *T. thermophila*-exposed *A. hydrophila***.

**Clones of different function**	**Locus tag**	**Gene name**	**Putative function**
**METABOLISM**			
Clone1	U876_00730		Aminotransferase
Clone2	U876_00745		Transporter
Clone3	U876_00975		Ornithine carbamoyltransferase
Clone4	U876_01225		Ktr system potassium transporter B
Clone5	U876_02130	*aceF*	Pyruvate dehydrogenase
Clone6	U876_03055		Nucleoside transporter NupC
Clone7	U876_04385		Nitrate ABC transporter ATP-binding protein
Clone8	U876_04680	*panF*	Sodium/panthothenate symporter
Clone9	U876_05745		Sodium:alanine symporter
Clone10	U876_05935	*astB*	N-succinylarginine dihydrolase
Clone11	U876_06095		Tungsten ABC transporter substrate-binding protein
Clone12	U876_06285		PTS system glucose-specific transporter subunit IIA
Clone13	U876_08200		Polyketide cyclase
Clone14	U876_08220	*gltD*	Glutamate synthase
Clone15	U876_08710	*oppA*	Peptide ABC transporter substrate-binding protein
Clone16	U876_10345		Acyl-coa dehydrogenase
Clone17	U876_10925		Chlorohydrolase
Clone18	U876_11870		Methylcitrate synthase
Clone19	U876_13100		2-oxoglutarate dehydrogenase
Clone20	U876_13390	*purF*	Amidophosphoribosyltransferase
Clone21	U876_14885	*napA*	Nitrate reductase
Clone22	U876_14960		Acyl-coa dehydrogenase
Clone23	U876_15035		Diguanylate phosphodiesterase
Clone24	U876_15360		Flavodoxin
Clone25	U876_15445		Arsenate reductase
Clone26	U876_16445		Formate acetyltransferase
Clone27	U876_16540		Peroxidase
Clone28	U876_17455		Na(+)-translocating NADH-quinone reductase subunit E
Clone29	U876_18130		Hypothetical protein
Clone30	U876_18245		3-oxoacyl-ACP synthase
Clone31	U876_19135		Methionine ABC transporter permease
Clone32	U876_19260		Acetolactate synthase 3 catalytic subunit
Clone33	U876_19385		Diguanylate phosphodiesterase
Clone34	U876_20360	*thyA*	Thymidylate synthase
Clone35	U876_20420		Transporter
Clone36	U876_20490		Nitrogen regulatory protein P-II 1
Clone37	U876_20575		Metallophosphatase
Clone38	U876_22695	*mgtA*	Magnesium ABC transporter atpase
Clone39	U876_22910	*cysE*	Serine acetyltransferase (cyse)
Clone40	U876_23285	*norV*	Nitric oxide reductase
Clone41	U876_23350		Thiosulfate sulfurtransferase
Clone42	U876_23375		3-octaprenyl-4-hydroxybenzoate carboxy-lyase
**CELLULAR PROCESSES AND SIGNALING**			
Clone43	U876_00105		Transporter
Clone44	U876_00195		Guanosine-3′,5′-bis(diphosphate) 3′-pyrophosphohydrolase
Clone45	U876_00655	*rstB*	Histidine kinase
Clone46	U876_05085	*dsbC*	Thiol:disulfide interchange protein DsbC
Clone47	U876_07030		Nucleoside-diphosphate sugar epimerase
Clone48	U876_08485		Glutathione s-transferase
Clone49	U876_03385	*msrB*	Methionine sulfoxide reductase b
Clone50	U876_09340	*msrA*	Methionine sulfoxide reductase a
Clone51	U876_09565		Alanine racemase
Clone52	U876_10180	*clpP*	Clp protease ClpP
Clone53	U876_10190	*lon*	DNA-binding protein
Clone54	U876_10495		Diguanylate cyclase
Clone55	U876_12865		Type I secretion protein
Clone56	U876_13410		Membrane protein
Clone57	U876_13455	*clpA*	Clp protease ClpA
Clone58	U876_16050		Glutathione s-transferase
Clone59	U876_17245	*rseP*	Zinc metallopeptidase rsep
Clone60	U876_17490		Lipoprotein
Clone61	U876_18695		Curculin (mannose-binding) lectin protein
Clone62	U876_19600	*ftsB*	Cell division protein ftsb
Clone63	U876_20365	*lgt*	Prolipoprotein diacylglyceryl transferase
Clone64	U876_22065		Preprotein translocase subunit
**INFORMATION STORAGE AND PROCESSING**			
Clone65	U876_00010	*dnaN*	DNA polymerase III subunit beta
Clone66	U876_01305	*rpoC*	DNA-directed RNA polymerase subunit beta
Clone67	U876_02985		integrase
Clone68	U876_04045		Aspartate aminotransferase
Clone69	U876_05075	*xerD*	Site-specific tyrosine recombinase xerd
Clone70	U876_07785		IS66 family element, transposase
Clone71	U876_08775		Integrase
Clone72	U876_09595		Chemotaxis protein
Clone73	U876_11735		Ribonuclease
Clone74	U876_14710		Restriction endonuclease subunit R
Clone75	U876_14980		DNA polymerase III subunit epsilon
Clone76	U876_15165		Translation elongation factor p (ef-p)
Clone77	U876_16880		Transcriptional regulator
Clone78	U876_17835	*radC*	DNA repair protein RadC
Clone79	U876_19085		tRNA dimethylallyltransferase
Clone80	U876_19280		TetR family transcriptional regulator
Clone81	U876_22665		XRE family transcriptional regulator
Clone82	U876_22995		LacI family transcriptional regulator
Clone83	U876_23655		Transcriptional antiterminator
Clone84	U876_24040		DNA helicase
**VIRULENCE FACTORS**			
Clone85	U876_00320	*bvgS*	Virulence sensor protein bvgs
Clone86	U876_02160		Serine protease
Clone87	U876_03105	*tapZ*	Pilus assembly protein tapz
Clone88	U876_03150	*cheX*	Chemotaxis protein chex
Clone89	U876_04005		RTX toxin
Clone90	U876_05350		Thermostable hemolysin
Clone91	U876_05485	*pilQ*	Pilus assembly protein pilq
Clone92	U876_07285	*flgH*	Flagellar L-ring protein flgh
Clone93	U876_08265	*rtxA*	Structural toxin protein rtxa
Clone94	U876_09965	*aroA*	3-phosphoshikimate 1-carboxyvinyltransferase
Clone95	U876_14270		Flagellin
Clone96	U876_16140	*cheW*	Chemotaxis protein chew
Clone97	U876_17740	*vgrG*	Rhs element Vgr family protein
Clone98	U876_17750	*hcp*	Hemolysin co-regulated protein
Clone99	U876_18290	*cheD*	Chemotaxis protein ched
Clone100	U876_20250		Type IV pilin
Clone101	U876_20280		Pilus assembly protein
Clone102	U876_21920	*mshL*	MSHA biogenesis protein MshL
**POORLY CHARACTERIZED**			
Clone103	U876_01055		Membrane protein
Clone104	U876_01550		Pirin
Clone105	U876_03465		Transporter
Clone106	U876_04000		Transporter
Clone107	U876_06575		Hypothetical protein
Clone108	U876_08425		Hydrolase
Clone109	U876_09200		Hypothetical protein
Clone110	U876_10290		Hypothetical protein
Clone111	U876_12075		Hypothetical protein
Clone112	U876_13245		Hypothetical protein
Clone113	U876_14160		Hypothetical protein
Clone114	U876_15330		Hypothetical protein
Clone115	U876_16040		Acetyltransferase
Clone116	U876_21535		Hypothetical protein

### Validation of SCOTS results by qRT-PCR

**Figure 4:**
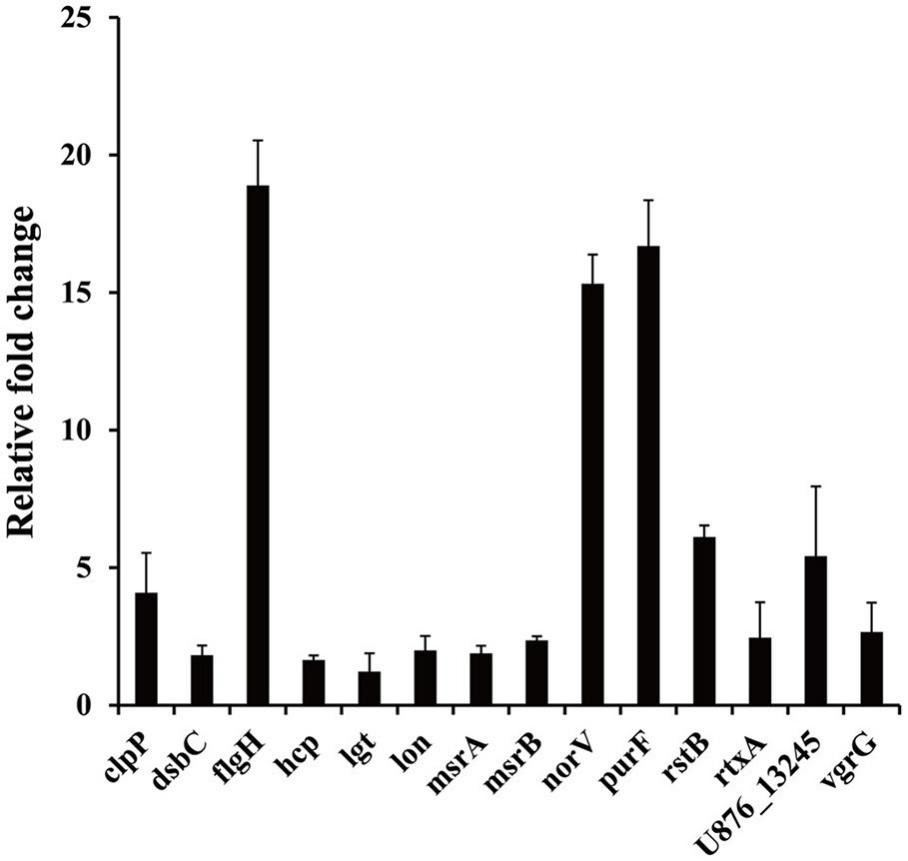
**Relative expression of genes in protozoa-exposed *A. hydrophila* compared to protozoa-unexposed *A. hydrophila***. Data are presented as relative fold changes with protozoa-unexposed *A. hydrophila* as the control and all fold changes are normalized to 16S rDNA. Relative fold changes were calculated using the 2^−ΔΔCt^ method, where ΔΔCt = (Ct_gene of interest_ − Ct_control gene_)_protozoa-exposed group_ − (Ct_gene of interest_ − Ct_control gene_)_protozoa-unexposed group_. Error bars represent standard deviations from three independent experiments.

The results of the SCOTS experiments were confirmed by qRT-PCR. Fourteen genes (*clpP, dsbC, flgH, hcp, lgt, lon, msrA, msrB, norV, purF, rstB, rtxA*, U876_13245, and *vgrG*) belonging to different functional categories were chosen and validated. As shown in Figure 4, compared with the protozoa-unexposed group, the expression levels of all 14 genes were up-regulated significantly in protozoa-exposed *A. hydrophila* except for *lgt* (1.22-fold change, *P* = 0.83). Among the remaining 13 genes, the expression levels of three genes *dsbC, hcp*, and *msrA* ranged from 1.64- to 1.89-fold (*P* < 0.05), whereas the expression levels of other 10 genes all changed more than two-fold (*P* < 0.05). The high coincidence rate (92.9%) of qRT-PCR with SCOTS indicates the reliability of the SCOTS results.

### Effect of *msr* inactivation on resistance of *A. hydrophila* to predation by *T. thermophila*

**Figure 5:**
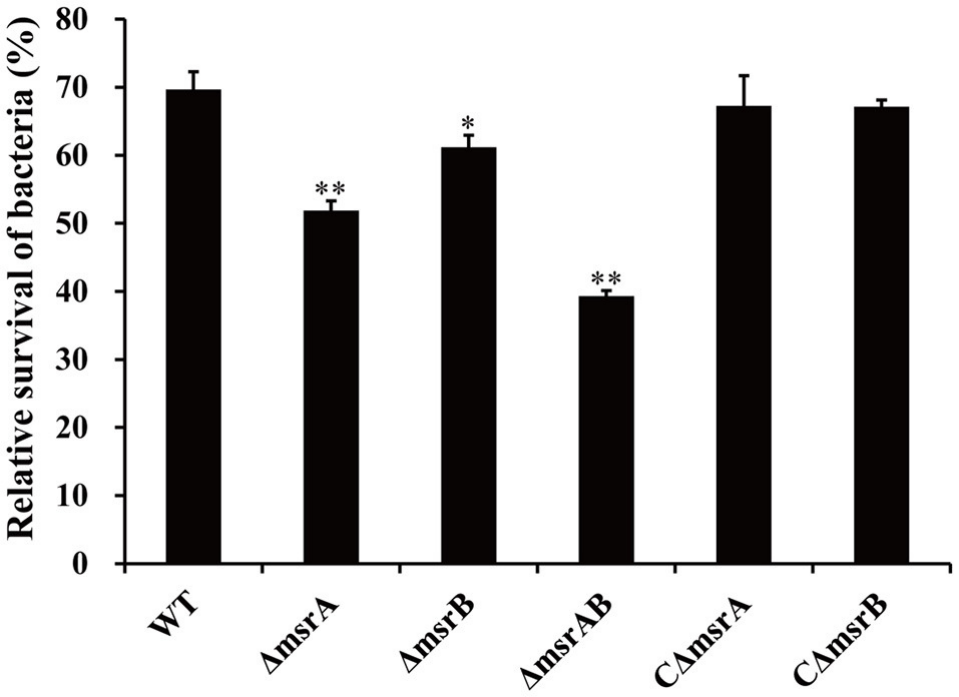
**Relative survival of a wild-type *A. hydrophila* and its *msr* gene mutant derivatives after co-culture with *T. thermophila***. WT represents the wild-type strain NJ-35. The relative survival of bacteria was expressed as the OD450 value of *A. hydrophila* co-cultured with *T. thermophila* divided by that of *A. hydrophila* grown alone at 12 h. The error bars represent standard deviations from four independent experiments performed in quadruplicate. ^*^P < 0.05 or ^**^P < 0.01 indicates significantly different relative survival compared with the WT group.

To further validate the SCOTS results and also determine whether *msrA* and *msrB* play important roles during co-culture of *A. hydrophila* strains with *T. thermophila*, the mutants Δ*msrA*, Δ*msrB*, and Δ*msrAB* were constructed by homologous replacement in strain NJ-35. The relative survivals of the wild-type and mutant strains after co-culture with *T. thermophila* are shown in Figure 5. Compared to the wild-type strain, the relative survivals of strains Δ*msrA* and Δ*msrB* were 17.77% lower (*P* < 0.01) and 8.46% lower (*P* < 0.05), respectively. Δ*msrAB* exhibited obviously lower relative survival (30.35%) than the wild-type strain (*P* < 0.01). However, the relative survivals of the complemented strains CΔ*msrA* and CΔ*msrB* were restored to the level of the wild-type strain. These results suggest that *msrA* and *msrB* play important roles in the resistance of *A. hydrophila* to protozoan predation.

### Effect of *msr* inactivation on the virulence of *A. hydrophila* in zebrafish

To investigate the roles of *msrA* and *msrB* in the virulence of *A. hydrophila*, zebrafish were injected intraperitoneally with the wild-type or mutant strains. The mortality of zebrafish was recorded daily over a period of 7 days following infection. As shown in Table 3, the LD_50_ value of the *msrA* mutant (7.68 × 10^1^ CFU) was nearly four-fold higher than that of the wild-type strain (2.05 × 10^1^ CFU), indicating a significant reduction in the virulence of the mutant. However, the LD_50_ value of the *msrB* mutant (1.89 × 10^1^ CFU) was similar to that of the wild-type strain, suggesting that *msrB* is not essential for the virulence of *A. hydrophila* in zebrafish. The simultaneous inactivation of *msrA* and *msrB* caused a more significant reduction in *A. hydrophila* virulence, and the LD_50_ value of the *msrAB* mutant (1.54 × 10^5^ CFU) was more than 2000-fold higher than that of the wild-type strain. These results suggest that MsrA plays an important role in the virulence of *A. hydrophila* in zebrafish and that a synergistic relationship may exist between MsrA and MsrB.

**Table 3:** **LD_50_ of the wild-type strain and msr genes mutants in zebrafish**.

**Bacteria/(CFU)**	**No. of zebrafish (Dead/Total)**			
	**NJ-35**	**Δ*msrA***	**Δ*msrB***	**Δ*msrAB***
10^7^	11/11	11/11	11/11	11/11
10^6^	11/11	11/11	11/11	7/11
10^5^	11/11	11/11	11/11	5/11
10^4^	11/11	10/11	11/11	2/11
10^3^	11/11	9/11	11/11	0/11
10^2^	10/11	7/11	9/11	0/11
10^1^	3/11	2/11	4/11	0/11
LD_50_	2.05 × 10^1^	7.68 × 10^1^	1.89 × 10^1^	1.54 × 10^5^

## Discussion

Hahn and Höfle (2001) reported that predation by protozoa can influence bacterial populations. Once preyed by protozoa, most microbes are digested as food, but some microbes appear to be resistant to protozoa digestion and can even replicate within protozoa. Several bacterial pathogens, including *E. coli* (King et al., 1988), *L. pneumophila* (Berk et al., 2008; Hojo et al., 2012), *S. enterica* (Brandl et al., 2005; Rehfuss et al., 2011), and *Listeria monocytogenes* (Pushkareva and Ermolaeva, 2010), have been shown to be resistant to destruction in digestive vacuoles of *Tetrahymena*. In this study, we observed that *T. thermophila* fed readily on *A. hydrophila* strains, however, LSCM and TEM observations and the survival rate of *A. hydrophila* in vacuoles indicated that the virulent strains were able to survive in *T. thermophila* vacuoles. Thus, *Tetrahymena* may represent an unappreciated reservoir for the hypervirulence phenotype of *A. hydrophila*. In this regard, previous reports have demonstrated that exposure to rumen protozoa leads to the selection of *Salmonella* strains with enhanced virulence traits (Rasmussen et al., 2005; Brewer et al., 2011). Therefore, protozoa may not only serve as a protective reservoir but also select for virulence traits.

We hypothesize that the survival of pathogenic *A. hydrophila* within *Tetrahymena* necessitates the expression of bacterial genes that are unlikely to be expressed in a protozoa-unexposed environment. In this study, 116 preferentially expressed genes were identified in *A. hydrophila* in response to phagocytosis by *Tetrahymena* using SCOTS. Genes involved in metabolism accounted for 36.2% (42/116) of differentially up-regulated genes in protozoa-exposed bacteria, including enzymes associated with amino acid transport, inorganic ion transport, energy production, carbohydrate transport, and metabolism. It is not surprising that *A. hydrophila* may alter its metabolism to obtain available nutrient and energy sources to adapt to the intracellular niche in *Tetrahymena*. Interestingly, some of these genes, including *panF, gltD, oppA, purF, napA, thyA, mgtA, cysE*, and *norV*, have been known to be associated with bacterial virulence or resistance in other bacteria. For instance, in *Moraxella catarrhalis*, an *oppA* mutant exhibited marked impairment in its capacity to persist in the respiratory tract compared to wild-type in a mouse pulmonary clearance model (Yang et al., 2011). Similarly, the mutation of the transport domain of the *oppA* gene in *Mycobacterium avium* resulted in bacterial attenuation in both macrophages and in mice (Danelishvili et al., 2014). The gene *purF*, which encodes amidophosphoribosyltransferase, was identified as a novel virulence factor in *Francisella tularensis* by screening a library of corresponding transposon mutants for replication in RAW264.7 macrophages (Llewellyn et al., 2011). In *Staphylococcus aureus*, inactivation of *thyA*, which is involved in thymidylate synthesis, strongly attenuated bacterial virulence in *Caenorhabditis elegans* and mouse models (Kriegeskorte et al., 2014). Another gene, *norV*, which encodes nitric oxide reductase, was observed to contribute to the survival of enterohemorrhagic *E. coli* (EHEC) O157 within macrophages (Shimizu et al., 2012). This obvious alteration of expression in these metabolism-related genes may be required for nutrient acquisition and virulence of *A. hydrophila* when exposed to *T. thermophila*.

In this study, 18 virulence-related genes were up-regulated in protozoa-exposed *A. hydrophila*. The structural toxin protein (RtxA) can disrupt the actin cytoskeleton of HeLa cells, resulting in a rounding phenotype and hence contributing to host cell apoptosis (Suarez et al., 2012). The gene *aroA* encodes 3-phosphoshikimate 1-carboxyvinyltransferase, and its inactivation has been reported to attenuate *A. hydrophila* virulence (Hernanz Moral et al., 1998; Vivas et al., 2004). In addition, Hcp and VgrG, two known T6SS effectors of *A. hydrophila*, were also identified in protozoa-exposed *A. hydrophila*. T6SS has been identified in 25% of sequenced Gram-negative genomes and is involved in virulence and host associations in these bacterial species (Pukatzki et al., 2007). Efficient colonization is critical for bacterial virulence, and both pili and flagella contribute to colonization in *A. hydrophila* (Tomas, 2012). In this study, genes responsible for the formation of type IV pili (*tapZ, pilQ*, and *mshL*) and flagella (*flgH*) were identified. Moreover, *cheX, cheW*, and *cheD*, which encode chemotaxis protein, were obtained using SCOTS. Antunez-Lamas et al. (2009) reported that the genes involved in the chemotactic signal transduction system and in the structure of the flagellar motor play important roles in the pathogenicity of *Dickeya dadantii*. In *A. hydrophila*, chemotaxis is not necessary for pathogenicity but may be a necessary parameter for this bacterium to become an obligate pathogen (Seshadri et al., 2006). The overall up-regulation of virulence genes in protozoa-exposed environments may explain why the virulent *A. hydrophila* strains had a greater ability to evade digestion by *T. thermophila*. Additionally, from an evolutionary perspective, the identification of the common virulence factors in protozoan and vertebrate hosts indicates the universality of virulence implicated in the infectious process in the evolutionarily divergent hosts.

Additionally, 22 genes, including *rstB, msrA, msrB, clpP*, and *clpA*, which are involved in cellular processes and signaling, were also identified. *RstB* encodes the sensor kinase and acts on the PhoQ sensor to control the expression of PhoP-regulated genes in *Salmonella* (Nam et al., 2010). The response regulator PhoP and its partner sensor PhoQ constitute the PhoP/PhoQ two-component system, which governs virulence, mediates the adaptation to Mg^2+^-limiting environments, and regulates other physiological processes of *Salmonella* (Groisman, 2001). Thus, RstB indirectly controls the virulence of *Salmonella*. The ATP-dependent caseinolytic proteases (Clp) are important in the resistance of pathogenic bacteria against environmental stresses and host immune defenses. ClpP is the proteolytic subunit, and ClpA acts as both a chaperone and an ATPase driving the degradation of damaged or improperly folded proteins. The *clpA* and *clpP* mutants of *Helicobacter pylori* exhibit increased sensitivity to oxidative stress, in addition to reduced survival in human macrophages (Loughlin et al., 2009). In addition, the ClpP protein is required for the stress tolerance of *Actinobacillus pleuropneumoniae* (Xie et al., 2013).

Methionine sulfoxide reductases (Msrs) are key enzymes in repairing ROS-mediated damage to proteins and include mainly MsrA and MsrB (Sansom et al., 2013). As the best characterized Msr, MsrA plays a role in resistance to oxidative stress and virulence in a number of bacteria, including *Mycobacterium* species (St. John et al., 2001; Douglas et al., 2004), *S. aureus* (Singh and Moskovitz, 2003), *Salmonella typhimurium* (Denkel et al., 2011), and *E. coli* (St. John et al., 2001). In this study, both *msrA* and *msrB* were up-regulated in *A. hydrophila* during co-culture with *T. thermophila*. To determine the role of the two genes in response to phagocytosis by *Tetrahymena*, we constructed the mutants Δ*msrA*, Δ*msrB*, and Δ*msrAB*. Single and double inactivation of *msrA* and *msrB* significantly reduced the resistance of *A. hydrophila* to predation by *T. thermophila*. These findings indicate that *msrA* and *msrB* were required for *A. hydrophila* to resist predatory protozoans. Moreover, the *msr* genes have previously been characterized as required for bacterial survival and replication within macrophages (Douglas et al., 2004; Sansom et al., 2013). These findings suggest that the mechanisms responsible for survival within the phagosomes of protozoa and macrophages may be similar. In addition, we observed that the deletion of *msrA* resulted in significantly reduced virulence in zebrafish, whereas the virulence of the *msrB* mutant was essentially unaffected. Notably, the double deletion of the *msr* genes (Δ*msrAB*) resulted in an extreme reduction of virulence (2000-fold higher LD_50_ value than Δ*msrA* strain), suggesting a synergistic effect of these two genes on bacterial virulence.

The present study is the first to characterize gene expression in *A. hydrophila* under phagocytosis by *Tetrahymena*. In this study, 116 genes were identified as up-regulated, including genes associated with metabolism, cellular process and signaling, information storage and processing, virulence factors, as well as some genes whose functions are currently unknown. Because protozoa share many features with mammalian phagocytes, particularly macrophages (Jacobs et al., 2006; Cosson and Soldati, 2008), a better understanding of protozoa-bacteria interactions will provide fascinating glimpses into host-pathogen relationships. This study will be a starting point for investigating the co-evolution of bacteria and protozoa. Future functional characterization of the genes identified in this study will deepen our understanding of the epidemiology of an infectious disease and the development of procedures for its control.

## Author contributions

YL, MP, and XL conceived the study and drafted the paper; MP, XL, and JL performed the experiments; CG, SG, and HD helped with the experiments; CG and CL provided valuable suggestions.

### Conflict of interest statement

The authors declare that the research was conducted in the absence of any commercial or financial relationships that could be construed as a potential conflict of interest.
