# Supplementary material for: Identification of Aeromonas hydrophila Genes Preferentially Expressed after Phagocytosis by Tetrahymena and Involvement of Methionine Sulfoxide Reductases
Source: Front Cell Infect Microbiol. 2016 Dec 26;6:199. doi: 10.3389/fcimb.2016.00199 (PMC5183988; doi:10.3389/fcimb.2016.00199)
Supplement: Supplementary file 1 [file Table1.DOC]

Supplementary Material

**Identification of *Aeromonas hydrophila* genes preferentially expressed after phagocytosis by *Tetrahymena* and involvement of methionine sulfoxide reductases**

Maoda Pang^1,2^, Xiaoqin Lin^1^, Jin Liu^1^, Changming Guo^1^, Shanshan Gao^1^, Hechao Du^1^, Chengping Lu^1^, Yongjie Liu^1*^

**Corresponding Author:** Yongjie Liu (liuyongjie@njau.edu.cn)

**Supplementary Table**

**Supplementary Table 1 Primers used in this study**

| **Primer** | **Nucleotide sequence (5’ to 3’)** | **Source** |
| --- | --- | --- |
| **Primer for SCOTS** |  |  |
| SCOTS-N6-01 | GACACTCTCGAGACATCACCGGTACCNNNNNN | This work |
| SCOTS-N6-02 | TGCTCTAGACGTCCTGATGGTTCNNNNNN | This work |
| SCOTS01 | GACACTCTCGAGACATCACCGGTACC | This work |
| SCOTS02 | TGCTCTAGACGTCCTGATGGTTC | This work |
| 16S01 | AGAGTTTGATCATGGCTCAGAT | This work |
| 16S02 | AAGGAGGTGATCCAACCCCAGG; amplify 16S rRNA (1537 bp) |
| 23S101 | GTTGTATGGTTAAGTGACTAAG | This work |
| 23S102 | GGCCCACCTAGCCTTCTCCGTC; amplify 5’ end of 23S rRNA (1440 bp) |
| 23S201 | GGTTAATATTCCTGCACGACTT | This work |
| 23S202 | TTCACGCTTAGATGCTTTCAGC; amplify 3’ end of 23S rRNA (1377 bp) |
| **Primer for qRT-PCR** |  |  |
| 16S rRNA-F | GTCTTGTAGAGGGGGGTA | This work |
| 16S rRNA-R | TGAGCGTCAGTCTTTGTC; 104 bp |
| *clpP*-F | CGATGTAGATGCTGATGT | This work |
| *clpP*-R | GCTGGAGGAGCGGGTGAT ; 122 bp |
| *dsbC*-F | ATCGGTAATTAAGCGGTT | This work |
| *dsbC*-R | CGTTGCTCTATGGTCTGC; 176 bp |
| *flgH*-F | ATATTGGCCTTCTTGGAGG | This work |
| *flgH*-R | ATCAGGTCAACGGCATCTA ; 103 bp |
| *hcp*-F | GTGTCCACAAGCCGTTCA | This work |
| *hcp*-R | GTACCACTTCAGGGTCACTTTC; 111 bp |
| *lgt*-F | CACCGGTCCAGATCTTG | This work |
| *lgt*-R | CTGTTTGGTTTCGCCTT; 205 bp |
| *lon*-F | CCGCTTGATACCACCGTG | This work |
| *lon*-R | CGCTATGTGTACCGCCCT; 154 bp |
| *msrA-*F | ACCCAGAATCCCACCTACAAG | This work |
| *msrA-*R | TTGAGCAAGTCGCCGTAGC; 107 bp |
| *msrB-*F | CGCAAGAGCGGCGACTAC | This work |
| *msrB-*R | CCTCGGTGTAGGTCACGGTC; 130 bp |
| *norV*-F | CAGCACTACTGCGACGAGC | This work |
| *norV*-R | GAAACCGAGCACTTCCTTGAT; 141 bp |
| *purF*-F | TGCCGTAAGGAGAGTT | This work |
| *purF*-R | ACAGGGGACAGGATGC; 210 bp |
| *rstB*-F | CCCCAACCCCTCTCCCGCA | This work |
| *rstB*-R | CAGCAACCCGACCCGCATC; 125 bp |
| *rtxA*-F | GCGACACCACCAATGC | This work |
| *rtxA*-R | AGCGGAACACGCCATT; 138 bp |
| U876_13245-F | TAGAGCTGGCCGAACAC | This work |
| U876_13245-R | GCAGACCGACTTTACCC; 129 bp |
| *vgrG*-F | TTGCCGTTCGGGTCAT | This work |
| *vgrG*-R | TTGCCGTTCGGGTCAT; 150 bp |
| **Primer for mutant construction** |  |  |
| *msrA-*up-F | CAGGTCGACTCTAGAGGATCCATGAGTTTGTCTGCCTTG (*Bam*H I) | This work |
| *msrA-*up-R | CGTCATCTTCACGCACCCTGTCCTTGTAT; 906 bp |
| *msrA-*down-F | AGGACAGGGTGCGTGAAGATGACGGGTATCTGT; 861 bp | This work |
| *msrA-*down-R | AGCTCGGTACCCGGGGATCCTTTACGGTATGGCTTTGAG (*Bam*H I) |
| *msrB-*up-F | CAGGTCGACTCTAGAGGATCCCAACAACAACGCCTACTGC (*Bam*H I) | This work |
| *msrB-*up-R | CGGGCGCCAGACACTGACTCCTTGCCGTGAAT; 669 bp |
| *msrB-*down-F | CAAGGAGTCAGTGTCTGGCGCCCGGCAT; 1024 bp | This work |
| *msrB-*down-R | GAGCTCGGTACCCGGGGATCCTCACCCTCCCCTCCTTCCT (*Bam*H I) |
| *msrA*-F | GAGCTCGGTACCCGGGGATCCAACAGCAATGGGCTCAAT | This work |
| *msrA*-R | CAGGTCGACTCTAGAGGATCCGCCCTGCTTGCTATCATT; 1129 bp |
| *msrB*-F | GAGCTCGGTACCCGGGGATCCTTCCTGCCCGACGACATC | This work |
| *msrB*-R | CAGGTCGACTCTAGAGGATCCTGGCCCCCACCGACCT; 816 bp |
